# Interpretation of DNA data within the context of UK forensic science — evaluation

**DOI:** 10.1042/ETLS20200340

**Published:** 2021-05-24

**Authors:** Roberto Puch-Solis, Susan Pope

**Affiliations:** 1 Aston University, Aston, U.K.; 2 Principal Forensic Services, Reading, U.K.

**Keywords:** DNA data, DNA profiling, forensic, interpretation

## Abstract

Forensic DNA provides a striking contribution to the provision of justice worldwide. It has proven to be crucial in the investigative phase of an unsolved crime where a suspect needs to be identified, e.g. from a DNA database search both nationally and internationally. It is also a powerful tool in the assignment of evidential weight to the comparison of a profile of a person of interest and a crime scene profile. The focus of this document is the evaluation of autosomal profiles for criminal trials in the UK. A separate review covers investigation and evaluation of Y-STR profiles, investigation using autosomal profiles, kinship analysis, body identification and Forensic Genetic Genealogy investigations. In less than 40 years, forensic DNA profiling has developed from a specialist technique to everyday use. Borrowing on advances in genome typing technology, forensic DNA profiling has experienced a substantial increase in its sensitivity and informativeness. Alongside this development, novel interpretation methodologies have also been introduced. This document describes the state of the art and future advances in the interpretation of forensic DNA data.

## Introduction

Forensic DNA is now a cornerstone of forensic science. Since its introduction into casework in the 1980s [1], it has benefited from a scientific basis in genetics for its production, and a probabilistic basis for its reporting. This document presents the state of the art in the UK and the future for the interpretation of DNA data within the context of forensic cases. The content of this topic is vast and therefore the review has been divided into two reviews. Here the focus is on the evaluation of autosomal DNA, i.e. assigning evidential value to a DNA profile from a crime scene and a profile from a person of interest (POI). A separate review covers the provision of intelligence for investigation (identifying suspects), evaluation of Y-STR profiles, kinship analysis, body identification and Forensic Genetic Genealogy investigations.

DNA data consists of (a) information provided in the production of a DNA profile, e.g. DNA quantity and percentage of contribution by donors; (b) contextual information of the case; and (c) external databases such as national DNA databases for investigation or allele frequency databases for assigning evidential weight.

Evaluation of DNA data provides powerful evidence to help address questions of the origin of the DNA in the sample and assists with the mechanism for deposition of DNA on an item. Interpretation of DNA data for evaluation is the assignment of probative value to DNA findings, consisting of DNA profiles from a POI and from a crime scene, when considering the findings under the views of the prosecution and the defence in a criminal case.

These findings are put into the context of the particular case circumstances so that the legal practitioners and jury can use this information in their deliberation giving it the right level of importance. It is performed with the application of the case assessment and interpretation (CAI) methodology. A central component of CAI is the use of Bayes’ theorem which has at its core the likelihood ratio (LR) as a way of assigning evidential weight to DNA findings.

## Provision of Forensic Services in the UK

There are three jurisdictions in the UK: England and Wales, Scotland and Northern Ireland. The forensic services are provided by police laboratories, private forensic providers, specialist companies and Universities, with quality being overseen by the Forensic Science Regulator of England and Wales, and procedures being accredited by the United Kingdom Accreditation Service (UKAS).

## From a crime scene to a DNA profile

Some regions of DNA contain information that codes for the production of the proteins that carry out functions within cells. Some DNA does not code for any protein and since the selection pressure on this is less than for the genes encoding proteins, more mutations can accumulate in these regions. In current forensic DNA analysis this variation in non-coding DNA is used to distinguish between individuals. The region consists of a short sequence of, usually, three to four nucleotides which are repeated a number of times, called Short Tandem Repeats (STR). The number of copies of repeated DNA is defined as an allele, e.g. allele 13 at DNA region (marker or locus) D18S51 has 13 copies of sequence AGAA and can be written as [AGAA]13.

**Figure 1.**
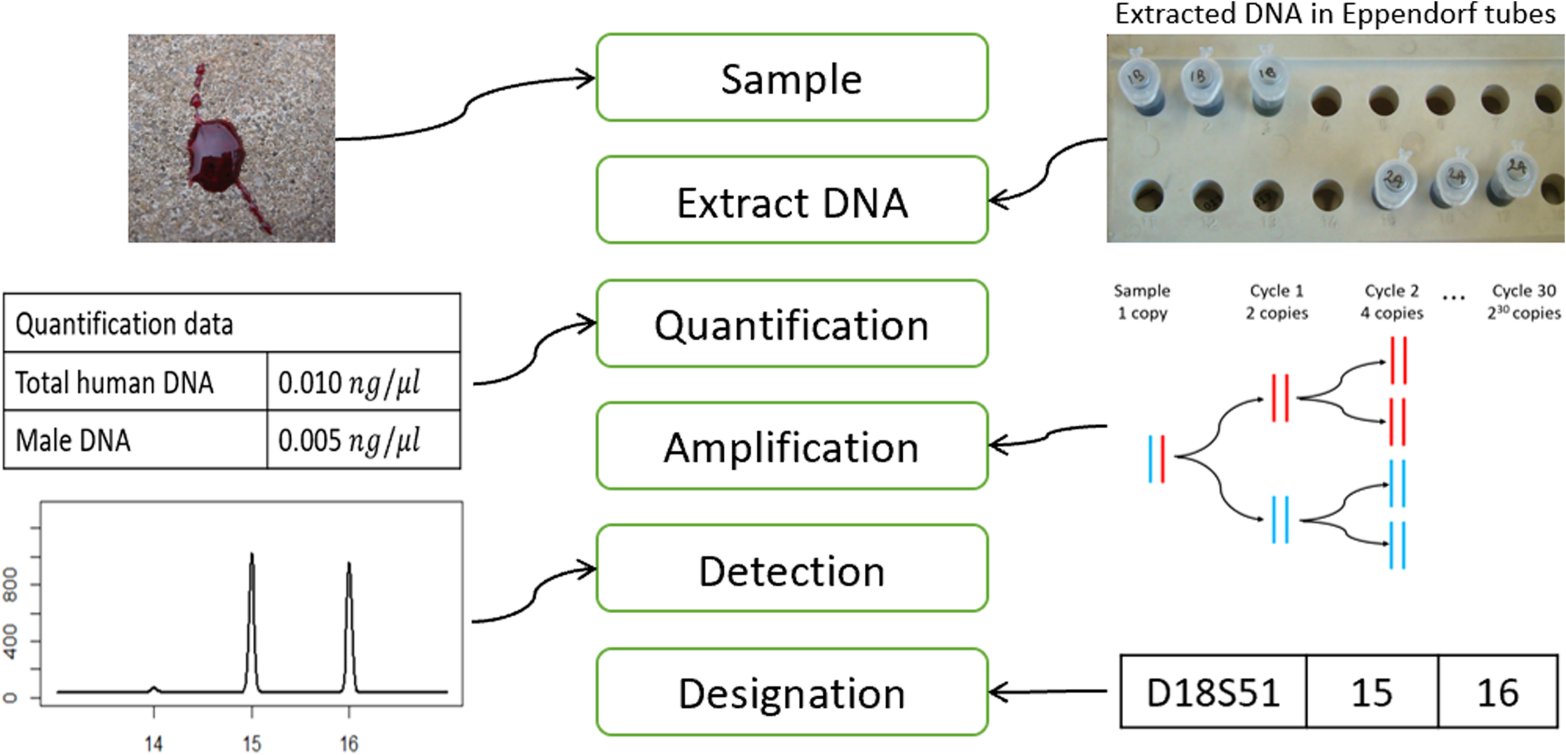
Diagrammatic representation of DNA analysis process from taking a sample to designating a profile. The rounded boxes represent the stages. The diagrams and pictures at each side illustrate the stages.

Figure 1 shows a diagrammatic description of the process of producing a DNA profile. DNA is extracted from items or samples and the amount recovered is quantified. The DNA is then divided into parts (aliquots) and amplified using polymerase chain reaction (PCR) to increase the amount of DNA to reach detectable levels. Several regions, usually called loci or markers, are tested.

**Figure 2.**
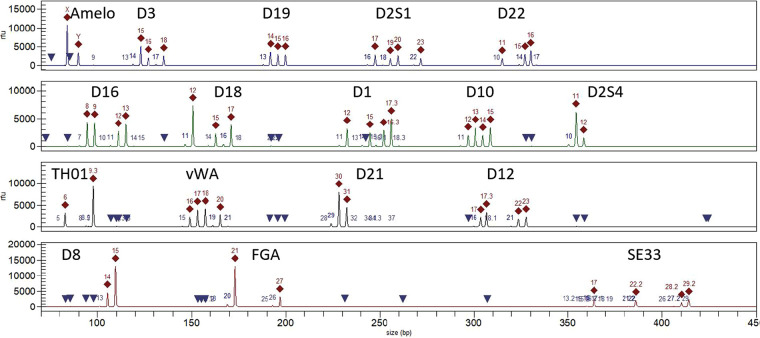
An epg of a balanced two-person mixture showing allele designations (red diamonds) and artefacts (blue triangles or blue numbers). The text labels are the locus names.

The amplified DNA is then separated by size using capillary electrophoresis to produce an electropherogram (epg), which consists of peaks where the x-axis location is the length of DNA expressed as the number of STR repeats within each specific region, and peak heights in the y-axis are roughly proportional to DNA quantity. Figure 2 shows an epg of an ESI profile, described in Table 1. Software is then used to designate allelic peaks and artefacts. The whole process from extraction to designation is largely automated apart from specialised extraction methods, e.g. those required to extract DNA from difficult samples like bone.

**Table 1:** Some current multiplexes

Locus	SGMPlus	NGMSE	ESI	DNA23+	CODIS expanded
D3	•	•	•	•	•
vWA	•	•	•	•	•
D16	•	•	•	•	•
D2S1	•	•	•	•	•
D8	•	•	•	•	•
D21	•	•	•	•	•
D18	•	•	•	•	•
D19	•	•	•	•	•
TH01	•	•	•	•	•
FGA	•	•	•	•	•
D1		•	•	•	•
D2S4		•	•	•	•
D10		•	•	•	•
D12		•	•	•	•
D22		•	•	•	•
SE33		•	•	•	
D7				•	•
CSF1PO				•	•
D13				•	•
TP0X					•
D5				•	•
Amelo	•	•	•	•	•
DYS391				•	•

A reference profile is produced from a sample taken from a known person under controlled conditions and with an optimal amount of DNA. Therefore, the designated profile, or genotype, provides all the information needed, see bottom of Figure 1. In contrast, an epg from a sample of questioned origin contains information, via peak heights, regarding several sources of uncertainty e.g. imbalanced between partner peaks, number of contributors, non-detected alleles (dropout), spurious peaks (dropin) and amount of DNA attributed to the contributors. Peak heights of alleles and some artefacts are used in the evaluation of data given hypotheses in relation to the origin of the DNA (see section titled “Sub-source”).

A DNA profile consists of a group of locus specific profiles. The set of combined loci are often called multiplexes. There are a variety of multiplexes in use across the world with an overlap between the DNA loci included in different multiplexes, Table 1. New multiplexes are back compatible with older multiplexes, e.g. SGMPlus, and cross compatible for international database searching, e.g. CODIS expanded. For an in depth treatment of this topic see [2]. Currently in the UK, NGMSE, ESI and DNA23+ are used routinely.

DNA analysis is undergoing major changes as a spin-off from methods developed by the human genome project. As a consequence, the DNA sequence of forensically important regions will be recorded rather than simply measuring the number of STRs (see section titled “Sub-source”). The new technology called Massively Parallel Sequencing (MPS) will also include regions for forensic DNA phenotyping, i.e. incorporating regions of DNA that are informative about the physical features of the donor of a profile [3].

## Interpretation foundation

Interpretation of DNA data in the UK and some parts of Europe follows the principles described in the CAI model [4–8]. The European Network of Forensic Science Institutes (ENFSI) supports this approach and provides guidelines for its application [9]. The Royal Statistical Society provides a set of four documents for guidance for Judges, Lawyers, Forensic Scientists and Expert Witnesses. One of them covers the CAI approach [10]. Recently, the forensic science regulator of England and Wales has published guidelines for the application of CAI across evidence types [11].

CAI promotes pre-assessment of potential findings as a way of mitigating cognitive bias [12]. CAI can be applied to an investigation, when a suspect needs to be identified based on a crime scene profile, and evaluation, when a suspect has been identified and the interest lies in how to assign the weight of evidence applied to the scientific findings.

A central concept for evaluation in CAI is that of propositions. In general, propositions can be classified as addressing the origin of the DNA (sub-source level [13], e.g. whether the DNA came from the POI), source of material (e.g. whether the semen came from POI), the activity (e.g. whether the POI had intercourse with the victim) and the offence level (e.g. whether the POI raped the victim). From this description it is clear that offence level is not in the remit of the forensic scientist.

Within CAI at least two mutually exclusive propositions must be considered. If the case circumstances, denoted here by *I*, do not provide information to formulate such propositions, it is not possible to evaluate the DNA findings. In criminal cases one proposition represents the view of the prosecution, *H*_1_, and the other that of the defence, *H*_2_ (see next section for some examples).

The logical approach for assigning a weight to DNA findings, denoted here by *E*, is based on Bayes’ theorem in its odds form [14],

$$\frac{\text{Pr}(H_{1}|I)}{\text{Pr}(H_{2}|I)}\;\times \;\;\frac{\text{Pr}(E|H_{1},I)}{\text{Pr}(E|H_{2},I)}\;\;=\;\;\frac{\text{Pr}(H_{1}|E,I)}{\text{Pr}(H_{2}|E,I)}.$$

Priorodds×likelihoodratio = posteriorodds

Expressed in words, the posterior odds are calculated as the prior odds multiplied by the LR. In the UK, for DNA at sub-source level, the jury uses its common sense to assign the prior odds and combine it with the LR, provided by the forensic scientist, to obtain the posterior odds, as defined in R v Doheny & Adams [15].

## Autosomal profile evaluation

At this stage there is a POI whose DNA profile has been produced from a reference sample taken from him/her. The task then is to evaluate whose DNA was present in the questioned sample and how it got there.

### Sub-source

The questioned sample may have come from one or more people. The complexities of calculating LRs considering all the sources of uncertainty, are similar. Therefore, in this section, we will consider a two-person mixture because it is rich enough to exemplify the features of mixture evaluation.

The court is interested in the origin of the DNA, which is addressed with a pair of propositions of the kind

*H*_1_: The DNA came from a POI and an unknown person unrelated to the POI

*H*_2_: The DNA came from two unknown people unrelated to each other and to the POI

An important aspect of the propositions is that they are posited based on the case circumstances and can be refined in the light of the DNA findings. The evaluation can be adapted if a different alternative proposition is put forward by the defence. The reappraisal of the propositions is ideally performed prior to the trial because it is time intensive and this allows the full process of statement writing, including peer review, to take place.

An LR considering a pair of propositions can be calculated using a probabilistic genotyping system (PGS), described below. For example, consider a case where the LR is greater than a billion. This is reported in a statement for court as:

*The DNA findings are at least a billion times more likely if H_1_ is true rather than H_2_ is true.*

Following FSR guidelines, LRs that are greater than one billion are reported in the UK as ‘at least one billion' [16]. There is a verbal scale [9,17] which is not intended for LRs calculated at the DNA sub-source level but for situations where no quantitative evaluation is possible. The International Society for Forensic Genetics (ISFG) has also produced guidelines [18].

A PGS combines the quality of the questioned profile and the rarity of the genotype pair combinations. The questioned profile consists of peak heights for each component detected. Weights, or probability densities, are assigned to the questioned profile assuming to have come from one putative genotype combination at a time from a large number of combinations. The rarity of a genotypes is assigned through a genotype probability, which requires a database of allele proportions published by the FSR of England and Wales [19], and stratified according to self-declared ethnicity [20]. It also requires allowances for both (a) the size of the allele proportion database and (b) subpopulation structure (shared distant ancestry) via a parameter usually called *θ* or *F*_ST_, [21].

Currently in the UK, there are two validated systems in routine use, a commercially available software, STRmix [22], and a proprietary system, LiRa [23]. Other systems are sometimes used such as freeware, DNAmixtures [24] EuroForMix [25] and likeLTD [26] and commercial software TrueAllele [27]. On rare occasions, such as for older unsolved (cold) cases, a discrete statistical model may be used for calculating an LR using LiRa discrete [28] or likeLTD discrete [29]. For a historic overview of PGS development see [30]. Guidelines for PGS validation has been produced in the UK [31], in Europe [32] and in the USA [33].

The calculation of an LR using a PGS requires entering the number of contributors, which is selected by the user. STRmix allows a range of values to be entered [34]. One approach is to estimate a probability distribution on the number of contributors using an external program [35,36] and make a decision on the value or values entered into the PGS [37]. Currently, PGSs can routinely perform LR calculations up to four person mixtures, however, some can perform five and six person mixture calculations.

For a more in depth description of evaluation of autosomal profile at sub-source level see [38–40].

In the future, for the specific task of addressing sub-source level propositions, STR regions will be typed down to base pairs, in addition to determining the number of repeats. Therefore, a single STR allele, e.g. allele 16, may have a number of variants resulting from differences in the base pair sequences. For example, allele 13 at DNA region D18S51 has at least two variants, variant 1301 has sequence [AGAA]13 and variant 1302 has sequence AGAA AGCA [AGAA]11 [41]. In 1301 the expression [AGAA]13 means that bases AGAA repeat 13 times, while in 1302 the second repeat is AGCA instead of AGAA followed by 11 repeats of AGAA.

There are several challenges for bringing MPS into practice. National databases will need to be updated to accommodate the additional variants, which requires careful consideration of internationally agreed nomenclature [42]. For the calculation of LRs, a database of allele counts, including the new variants, needs to be collated for each ethnic appearance populations within countries. Some work has been carried out already towards this goal [41]. A third challenge is to perform research on the behaviour of peak heights and artefacts when considering allele variants. Some research has been published to cover these aspects [42,43]. The first case using MPS has been reported by Peter de Knijff from Leiden University (Pers. Comm. P. de Knijff).

### Source

Sub-source level considers the origin of the DNA result while source adds the attribution of the DNA to a body fluid. Currently in the UK, subjective opinion of attributions is reported based on presumptive chemical tests, microscopy or visual appearance. Although there is an implicit pair of propositions, it is often expressed as, e.g. ‘In my opinion, the male DNA component of the result can be attributed to semen'

A review of body fluid identification method is given in [44]. DNA and RNA based methods are being developed. Some of these are in current use for casework at the Netherlands Forensic Institute (NFI) [45].

In the near future, LRs will be calculated for source level propositions at the NFI [46]. Incorporation of DNA and RNA based tests within MPS opens the possibility of calculating LRs that incorporate source and sub-source evaluation together.

### Activity

An important question for the court is the activity by which and the time when a DNA stain was left on an item of interest. At present in the UK, a forensic scientist reports these as a subjective opinion, e.g. ‘Based on my experience, the DNA results are what I would expect if the suspect had penetrated the victim. If an alternative version is provided, then I will reappraise my opinion'. Sometimes, a subjective opinion is aided by propositions, i.e.

*H*_1_: The suspect penetrated the victim, or

*H*_2_: The suspect held hands with the victim,

and reported as ‘*The DNA findings provide support for H*_1_ *rather than H*_2_'.

The opinions are based on a number of studies addressing activities for deposition of DNA which consider Transfer, Persistence, Prevalence and Recovery (TPPR) [47]. These measure background DNA, i.e. the pre-existing DNA unrelated to the incident which is found on surfaces. Transfer refers to the direct, or primary, and indirect, or secondary, deposition of DNA to items. Tertiary deposition may also be considered. Persistence refers to the retention and loss of DNA through other activities such as movement or washing. Recovery refers to locating the stain to be sampled and the efficiency of extracting DNA for analysis.

Publications on TPPR are very varied in terms of multiplexes, testing methods, factors that affect TPPR leaving forensic scientists with the challenging task of making sense of the literature and apply it to their case. Publications are sometimes gathered into databases, e.g. DNATrAC [48], to make the literature more accessible.

There is research and development exploring the use of Bayesian networks (BNs) for the evaluation of activity level propositions, see [49] for a review and [50] for some examples. To date, BNs have been used only to underpin an opinion which is then reported in court. It has taken some years from the first uses of BNs in forensic science [51,52] to reach a point where they are ready to support casework.

The Netherlands Register of Court Experts (Nederlands Register Gerechtelijk Deskundigen, NRGD) has recently introduced guidelines for registering forensic scientists who report activity level propositions to court [53]. The ISFG has also produced guidelines [54]. The FSR has published generic guidelines that are applicable to evaluative opinions that include activity level propositions [11]. A review of transfer research is given in [55]. Limitations of expert opinions is discussed in [56].

We envisage that in the future, probabilistic support systems (PSSs) will be developed to assist the forensic scientist to report activity level propositions. These will be underpinned by statistical models informed by data that can also incorporate expert knowledge. A PSS would be used for training, calibration of expert opinions and reporting. The statistical models and expert opinion would inform nodes in BNs, which would be an intrinsic part of a PSS.

The development of these systems would require fundamental experiments and observational studies on TPPR performed in following a systematic and structure methodology that will allow the produced data to be used by several organisations. Past initiatives have proposed these ideas [57].

## Summary

- In forensic science, DNA data is interpreted to provide assistance to criminal courts in determining whether the DNA of a person of interest is present in a crime stain and how and when it was deposited.
- Case Assessment and Interpretation gives a structure that clarifies the interpretation requirements and mitigates against cognitive bias.
- Probabilistic genotyping systems address whether the DNA originated from the person of interest.
- The amount and type of data produced for a DNA profile will increase with the implementation of Massively Parallel Sequencing (MPS) methods.
- The quantitative evaluation of source and activity level propositions by different experts will be calibrated and aided by Bayesian Networks and Probabilistic Support Systems.

## Open Access

Open access for this article was enabled by the participation of Aston University in an all-inclusive *Read & Publish* pilot with Portland Press and the Biochemical Society under a transformative agreement with JISC.

## Abbreviations

BNs: Bayesian networks
CAI: case assessment and interpretation
ISFG: International Society for Forensic Genetics
LR: likelihood ratio
MPS: Massively Parallel Sequencing
NFI: Netherlands Forensic Institute
PGS: probabilistic genotyping system
POI: person of interest
STR: Short Tandem Repeats
TPPR: Transfer, Persistence, Prevalence and Recovery

## References

[1] Gill, P., Jeffreys, A. and Werrett, D. (1985) Forensic application of DNA ‘fingerprints’. Nature 318, 577–579 10.1038/318577a0 3840867

[2] Butler, J. (2015) Advanced Topics in Forensic DNA Typing, Interpretation, Academic press, Oxford

[3] Palencia-Madrid, L., Xavier, C., de la Puente, M., Hohoff, C., Phillips, C., Kayser, M. et al. (2020) Evaluation of the VISAGE basic tool for appearance and ancestry prediction using PowerSeq chemistry on the MiSeq FGx system. Genes 11, 708–719 10.3390/genes11060708 PMC7349024 32604780

[4] Cook, R., Evett, I., Jackson, G., Jones, P. and Lambert, J. (1998) A hierarchy of propositions; deciding which level to address in casework. Sci. Justice 38, 231–239 10.1016/S1355-0306(98)72117-3

[5] Cook, R., Evett, I., Jackson, G., Jones, P. and Lambert, J. (1998) A model for case assessment and interpretation. Sci. Justice 38, 151–156 10.1016/S1355-0306(98)72099-4 9800430

[6] Evett, I., Jackson, G. and Lambert, J. (2000) More on the hierarchy of propositions; exploring the distinction between explanations and propositions. Sci. Justice 40, 3–10 10.1016/S1355-0306(00)71926-5 10795422

[7] Evett, I., Jackson, G., Lambert, J. and McCrossan, S. (2000) Impact of the principles of evidence interpretation on the structure and content of statements. Sci. Justice 40, 233–239 10.1016/S1355-0306(00)71993-9 11094820

[8] Jackson, G., Jones, S., Booth, G., Champod, C. and Evett, I. (2006) The nature of forensic science opinion, a possible framework to guide thinking and practice in investigations and in court proceedings. Sci. Justice 46, 33–44 10.1016/S1355-0306(06)71565-9 16878783

[9] ENFSI. (2015) ENFSI Guideline for Evaluative Reporting in Forensic Science, ENFSI. http://enfsi.eu/wp-content/uploads/2016/09/m1_guideline.pdf

[10] Jackson, G., Aitken, C. and Roberts, P. (2015) Case Assessment and Interpretation of Expert Evidence, Royal Statistical Society. https://rss.org.uk/RSS/media/File-library/Publications/rss-case-assessment-interpretation-expert-evidence.pdf

[11] Forensic Science Regulator. (2021) FSR-C-118 Development of Evaluative Opinions, Forensic Science Regulator, London

[12] Dror, I. (2020) Cognitive and human factors in expert decision making: six fallacies and the eight sources of bias. Anal. Chem. 92, 7998–8004 10.1021/acs.analchem.0c00704 32508089

[13] Buckleton, J., Triggs, C. and Walsh, S. (2005) Forensic DNA Evidence Interpretation, CRC Press

[14] Evett, I. and Weir, B. (1998) Interpreting DNA Evidence, Sinauer associates Inc

[15] Court of Appeal Criminal Division. (1996) British and Irish legal information institute (BAILII). Available from: http://www.bailii.org/ew/cases/EWCA/Crim/1996/728.html

[16] Forensic Science Regulator. (2020) FSR-G-213 Allele frequency databases and reporting guidance for the DNA (short tandem repeat) profiling, Available from: https://assets.publishing.service.gov.uk/government/uploads/system/uploads/attachment_data/file/915100/FSR-G-213_DNA_profile_interpretation_Issue_2_Final.pdf

[17] Association of Forensic Science Providers. (2009) Standards for the formulation of evaluative forensic science expert opinion. Sci. Justice 49, 161–164 10.1016/j.scijus.2009.07.004 19839414

[18] Gill, P., Hicks, T., Butler, J., Connolly, E., Gusmão, L., Kokshoorn, B. et al. (2018) DNA commission of the International society for forensic genetics: assessing the value of forensic biological evidence: guidelines highlighting the importance of propositions. Part I: evaluation of DNA profiling comparison given (sub)-source propositions. Forensic Sci. Int. Genet. 36, 189–202 10.1016/j.fsigen.2018.07.003 30041098

[19] Forensic Science Regulator. (2014) DNA population data to support the implementation of national DNA database DNA 17 profiling. Available from: https://data.gov.uk/dataset/c7d4178d-999b-41fd-9312-5399d2aff57d/dna-population-data-to-support-the-implementation-of-national-dna-database-dna-17-profiling

[20] Steele, C. and Balding, D. (2014) Choice of population database for forensic DNA profile analysis. Sci. Justice 54, 487–493 10.1016/j.scijus.2014.10.004 25498938 PMC4275602

[21] Steele, C., Syndercombe-Court, D. and Balding, D. (2014) Worldwide Fst estimates relatives to five continental scale populations. Ann. Hum. Genet. 78, 468–477 10.1111/ahg.12081 26460400 PMC4223938

[22] Taylor, D., Bright, J. and Buckleton, J. (2013) The interpretation of single source and mixed DNA profiles. Forensic Sci. Int. Genet. 7, 516–528 10.1016/j.fsigen.2013.05.011 23948322

[23] Puch-Solis, R., Clayton, T. and Barron, M. (2019) Research Gate. Available from: https://www.researchgate.net/publication/337290115_Evidential_evaluation_of_DNA_profiles_using_a_continuous_model_implemented_in_the_DNA_LiRa_software

[24] Graversen, T. and Lauritzen, S. (2014) Computational aspects of DNA mixture analysis. Stat. Comput. 25, 527–541 10.1007/s11222-014-9451-7

[25] Bleka, O., Storvik, G. and Gill, P. (2016) Euroformix: an open source software based on a continuous model to evaluate STR DNA profiles from a mixture of contributors with artefacts. Forensic Sci. Int. Genet. 21, 35–44 10.1016/j.fsigen.2015.11.008 26720812

[26] Steele, C., Greenhalgh, M. and Balding, D. (2016) Evaluation of low-template DNA profiles using peak heights. Stat. Appl. Genet. Mol. Biol. 15, 431–445 10.1515/sagmb-2016-0038 27416618

[27] Perlin, M., Legler, M., Spencer, C., Smith, J., Allan, W., Belrose, J. et al. (2011) Validating TrueAllele DNA mixture interpretation. J. Forensic Sci. 56, 1430–1447 10.1111/j.1556-4029.2011.01859.x 21827458

[28] Puch-Solis, R. and Clayton, T. (2014) Evidential evaluation of DNA profiles using a discrete statistical model implemented in the DNA liRa software. Forensic Sci. Int. Genet. 11, 220–228 10.1016/j.fsigen.2014.04.005 24815372

[29] Balding, D. (2013) Evaluation of mixed source low-template DNA profiles in forensic science. Proc. Natl Acad. Sci. U.S.A. 110, 12241–12246 10.1073/pnas.1219739110 23818643 PMC3725068

[30] Coble, M. and Bright, J. (2019) Probabilistic genotyping software: an overview. Forensic Sci. Int. Genet. 38, 219–224 10.1016/j.fsigen.2018.11.009 30458407

[31] Forensic Science Regulator. (2018) FSR-G-223 Software Validation for DNA Mixture Interpretation, Forensic Science Regulator, London

[32] Coble, M., Buckleton, J., Butler, J., Egeland, T., Fimmers, R., Gill, P. et al. (2016) DNA commission of the international society for forensic genetics: recommendations on the validation of software programs performing biostatistical calculations for forensic genetics applications. Forensic Sci. Int. Genet. 25, 191–197 10.1016/j.fsigen.2016.09.002 27643465

[33] SWGDAM. (2015) Guidelines for the Validation of Probabilistic Genotyping Systems, SWGDAM, Washington DC

[34] McGovern, C., Cheng, K., Kelly, H., Ciecko, A., Taylor, D., Buckleton, J. et al. (2020) Performance of a method for weighting a range in the number of contributors in probabilistic genotyping. Forensic Sci. Int. Genet. 48, 102352 10.1016/j.fsigen.2020.102352 32707473

[35] Benschop, C., van der Linden, J., Hoogenboom, J., Ypma, R. and Haned, H. (2019) Automated estimation of the number of contributors in autosomal short tandem repeat profiles using a machine learning approach. Forensic Sci. Int. Genet. 43, 102150 10.1016/j.fsigen.2019.102150 31476660

[36] Grgicak, M., Karkar, S., Yearwood-Garcia, X., Alfonse, L., Duffy, K. and Lun, D. (2020) A large-scale validation of NOCIT's a posteriori probability of the number of contributors and its integration into forensic interpretation pipelines. Forensic Sci. Int. Genet. 47, 102296 10.1016/j.fsigen.2020.102296 32339916

[37] Benschop, C., Hoogenboon, J., Hovers, P., Slagter, M., Kruise, D., Parag, R. et al. (2019) DNAxs/DNAStatistX; development and validation of a software suite for the data management and probabilistic interpretation of DNA profiles. Forensic Sci. Int. Genet. 42, 81–89 10.1016/j.fsigen.2019.06.015 31254947

[38] Puch-Solis, R., Roberts, P., Pope, S. and Aitken, C. (2012) Assessing the Probative Value of DNA Evidence, Royal Statistical Society. https://rss.org.uk/RSS/media/News-and-publications/Publications/Reports%20and%20guides/rss-assessing-probative-value.pdf

[39] Balding, D. and Steele, C. (2015) Weight-of-Evidence for Forensic DNA Profiles, Wiley, Chichester

[40] Buckleton, J., Bright, J. and Taylor, D. (2016) Forensic DNA Evidence Interpretation, 2nd edn, CRC press, Boca Raton

[41] Devesse, L., Davenport, L., Borsuk, L., Gettings, K., Mason-Buck, G., Vallone, P. et al. (2020) Classification of STR allelic variation using massively parallel sequencing and assessment of flanking region power. Forensic Sci. Int. Genet. 48, 102356 10.1016/j.fsigen.2020.102356 32712568

[42] Bleka, O., Just, J., Le, J. and Gill, P. (2020) An examination of STR nomenclatures, filters and models for MPS mixture interpretation. Forensic Sci. Int. Genet. 48, 102319 10.1016/j.fsigen.2020.102319 32563046

[43] Cheng, K., Skillman, J., Hickey, S., Just, R., Moreno, L., Bright, J. et al. (2020) Variability and additivity of read counts for aSTRs in NGS DNA profiles. Forensic Sci. Int. Genet. 48, 102351 10.1016/j.fsigen.2020.102351 32682320

[44] Harbison, S. and Fleming, R. (2016) Forensic body fluid identification: state of the art. Res. Rep. Forensic Med. Sci. 6, 11–23 10.2147/RRFMS.S57994

[45] Lindenbergh, A., Maaskant, P. and Sijen, T. (2013) Implementation of RNA profiling in forensic casework. Forensic Sci. Int. Genet. 7, 159–166 10.1016/j.fsigen.2012.09.003 23036470

[46] Ypma, R., Maaskant-van Wijk, P., Gill, R. and Sjerps, M. (2021) Calculating LRs for presence of body fluids from mRNA assay data in mixtures. Forensic Sci. Int. Genet. 52, 102455 10.1016/j.fsigen.2020.102455 33461104

[47] van Oorschot, R., Szkuta, B., Ballantyne, K. and Goray, M. (2017) Need for dedicated training, competency assessment, authorisations and ongoing proficiency testing for those addressing DNA transfer issues. Forensic Sci. Int. Genet. Suppl. Ser. 6, e32–e34 10.1016/j.fsigss.2017.09.013

[48] Gosch, A. and Courts, C. (2019) On DNA transfer: the lack and difficulty of systematic research and how to do it better. Forensic Sci. Int. Genet. 40, 24–36 10.1016/j.fsigen.2019.01.012 30731249

[49] Taylor, D., Kokshoorn, B. and Biedermann, A. (2018) Evaluation of forensic genetics findings given activity level propositions: a review. Forensic Sci. Int. Genet. 36, 34–49 10.1016/j.fsigen.2018.06.001 29929059

[50] Taylor, D., Samie, L. and Champod, C. (2019) Using Bayesian networks to track DNA movement through complex transfer scenarios. Forensic Sci. Int. Genet. 42, 69–80 10.1016/j.fsigen.2019.06.006 31234042

[51] Curran, J., Triggs, C., Buckleton, J., Walsh, K. and Hicks, T. (1998) Assessing transfer probabilities in a Bayesian interpretation of forensic glass evidence. Sci. Justice 38, 15–21 10.1016/S1355-0306(98)72068-4 9624809

[52] Aitken, C. and Gammerman, A. (1989) Probabilistic reasoning in evidential assessment. J. Forensic Sci. Soc. 29, 303–316 10.1016/S0015-7368(89)73270-9

[53] NRGD. (2020) DNA Analysis and Interpretation, Nederlands Register Gerechtelijk Deskundigen. https://english.nrgd.nl/registration/DNA-analysis-and-interpretation.aspx

[54] Gill, P., Hicks, T., Butler, J., Connolly, E., Gusmão, L., Kokshoorn, B. et al. (2020) DNA commission of the International society for forensic genetics: assessing the value of forensic biological evidence: guidelines highlighting the importance of propositions. Part II: evaluation of biological traces considering activity level propositions. Forensic Sci. Int. Genet. 44, 102186 10.1016/j.fsigen.2019.102186 31677444

[55] van Oorschot, R., Szkuta, B., Meakin, G., Kokshoorn, B. and Goray, M. (2019) DNA transfer in forensic science: a review. Forensic Sci. Int. Genet. 38, 140–166 10.1016/j.fsigen.2018.10.014 30399535

[56] Meakin, G., Kokshoorn, B., van Oorschot, R. and Szkuta, B. (2020) Evaluating forensic DNA evidence: connecting the dots. WIREs Forensic Sci., e1404 10.1002/wfs2.1404

[57] Curran, J. (2009) Use of knowledge based systems in forensic science. In Wiley Encyclopedia of Forensic Science (Jamieson, A. and Moenssens, A., eds), Wiley, Chichester

